# Down-Regulation of *SlGRAS10* in Tomato Confers Abiotic Stress Tolerance

**DOI:** 10.3390/genes12050623

**Published:** 2021-04-22

**Authors:** Sidra Habib, Yee Yee Lwin, Ning Li

**Affiliations:** 1 Key Laboratory of Plant Hormones and Development Regulation of Chongqing, School of Life Sciences, Chongqing University, Chongqing 400044, China; yeeyeelwinbot@gmail.com; 2 College of Life Sciences, Henan Normal University, Xinxiang 453007, China; lining2310@163.com

**Keywords:** *SlGRAS10*, abiotic stress, tolerance, tomato, ROS scavenging system, flavonoid

## Abstract

Adverse environmental factors like salt stress, drought, and extreme temperatures, cause damage to plant growth, development, and crop yield. GRAS transcription factors (TFs) have numerous functions in biological processes. Some studies have reported that the GRAS protein family plays significant functions in plant growth and development under abiotic stresses. In this study, we demonstrated the functional characterization of a tomato *SlGRAS10* gene under abiotic stresses such as salt stress and drought. Down-regulation of *SlGRAS10* by RNA interference (RNAi) produced dwarf plants with smaller leaves, internode lengths, and enhanced flavonoid accumulation. We studied the effects of abiotic stresses on RNAi and wild-type (WT) plants. Moreover, *SlGRAS10*-RNAi plants were more tolerant to abiotic stresses (salt, drought, and Abscisic acid) than the WT plants. Down-regulation of *SlGRAS10* significantly enhanced the expressions of catalase (CAT), peroxidase (POD), and superoxide dismutase (SOD) to reduce the effects of reactive oxygen species (ROS) such as O^2−^ and H_2_O_2_. Malondialdehyde (MDA) and proline contents were remarkably high in *SlGRAS10*-RNAi plants. Furthermore, the expression levels of chlorophyll biosynthesis, flavonoid biosynthesis, and stress-related genes were also enhanced under abiotic stress conditions. Collectively, our conclusions emphasized the significant function of *SlGRAS10* as a stress tolerate transcription factor in a certain variety of abiotic stress tolerance by enhancing osmotic potential, flavonoid biosynthesis, and ROS scavenging system in the tomato plant.

## 1. Introduction

Transcription factors are involved in plant growth. Among them, transcription factors from the GRAS family were recognized based on DNA binding, transcriptional activation features, and nuclear localization [1]. The GRAS plant proteins are important for controlling several features of development, growth, and retorts to the abiotic and biotic conditions. The GRAS protein family name derived from its major three known participants, i.e., gibberellic acid insensitive (GAI), the repressor of GA1 (RGA), and a scarecrow (SCR) [2,3]. Moreover, SCL14, a GRAS protein is known to be important for the stimulation of stress-inducible promoters in *Arabidopsis thaliana* [4].

The GRAS protein family is different from other proteins that usually have five conserved motifs in the C-terminus [3]. The functions of all GRAS proteins have not been characterized but usually, GRAS proteins function as transcription factors. A few years ago, several transcription factors have been recognized as fundamental members in controlling plant retorts to stresses [5]. For example, CBF and DREB proteins from the AP2 transcription factors attach with the dehydration response element and regulate the expression of stress-responsive genes [6]. High salinity and drought are the main stress elements persuading adverse effects, including plant development, inhibition of seed germination, and reduced fruit production. Several stress-related genes are persuaded in plants mainly controlled by a series of transcription factors under salt and drought stress [7]. GRAS proteins play a significant function under biotic and abiotic stresses in grape ripening [8]. The GRAS family genes play several functions in plant development and growth including gibberellins signal transduction [9,10], phytochrome A signal transduction [11], root radial organization [12], shoot meristem maintenance [13], male gametogenesis [1], and axillary meristem initiation [14,15]. GRAS family has also been related to abiotic stress response and plant disease resistance [16,17,18]. Suppression of *SlGRAS6* tomato plants indicated enhances disease resistance [16]. In rice, over-expression of *OsGRAS23* enhanced oxidative and drought stress tolerance [19]. In *Arabidopsis*, Populus gene *PeSCL7* ectopic expression increased salt and drought tolerance [17]. In *Arabidopsis*, a GRAS transcription factor *PAT1* (*Vitis amurensis*), overexpressing plants conferred more drought, cold, and salinity tolerance [20]. In crops like tomato, analyzing the functions of more GRAS genes will support to explicate the mechanisms regulating stress tolerance and probably help the breeding of tolerant accessions.

Tomato (*Solanum lycopersicum*) is responsive to a series of environmental stresses. Salinity and drought are key causes of stress that result in deleterious effects, such as decreased plant growth, inhibition of seed germination, and reduced fruit production. A significant study has been shown to understand the physiological characteristics of plants in response to salinity and provide sources to grow salinity-tolerant accessions for plant breeders [21]. In tomato, genome-wide analysis of GRAS proteins has been accomplished, and 53 members of GRAS proteins were recognized [22]. However, the role of several GRAS proteins is still unknown in tomatoes. Previously, our study discussed that *SlGRAS7* overexpressing plants showed pleiotropic phenotypes, including dwarf plant height, delay flower time, reduced stem diameter, and many others [23]. Furthermore, *SlGRAS7* overexpressing plants could enhance tomato tolerance under drought and salt environments [23]. Several functions of GRAS proteins have been described in other plants; it is significant to examine the roles of the remaining GRAS protein family in the tomato plant. In this research, we described the functional characterization of *SlGRAS10* (accession number: Solyc03g025170.1.1), which is from the subfamily of PAT1 protein. To learn about the evolutionary relationship of the *SlGRAS10* and other organisms, we had constructed a phylogenetic tree of *SlGRAS10* and other plant proteins based on the peptide sequences, indicating that the *AtSCL8* is the closest orthologous to *SlGRAS10*. In our study, expression of *SlGRAS10* showed higher levels in leaves and red fruit in WT. Moreover, the transcript accumulation of *SlGRAS10* sharply enhanced under salt stress, dehydration, wounding and might be caused by exogenous use of GA_3_, Abscisic acid (ABA), and ACC. To further study the role of *SlGRAS10*, we constructed an RNAi vector to produce *SlGRAS10* down-regulated transgenic lines which have been resulted in pleiotropic phenotypes. To examine whether down-regulation of *SlGRAS10* plants confers abiotic stress tolerance, the *SlGRAS10*-RNAi transgenic plants treated with salt and drought stress. In present work, expression of *SlGRAS10* was remarkably higher under abiotic stress has been determined. Generally, the drought stress and ABA signaling share the same components, so we further observed the *SlGRAS10*-RNAi transgenic plants treated with ABA stress. Our study examined that the down-regulation of *SlGRAS10* plants increased tolerance under abiotic stress by modulating the stress-inducible genes expression that are intricate in physiological modifications, such as flavonoid biosynthesis, reactive oxygen species (ROS) scavenging system, chlorophyll biosynthesis pathway and senescence associated genes. We consider that this study might deliver a different vision of the functional characterization of the *GRAS* gene family to abiotic stress resistance in tomato and different plant species.

## 2. Materials and Methods

### 2.1. Phylogenetic Analysis

For phylogenetic tree analysis, peptide sequences of 14 other GRAS family proteins from other plant species were selected and a dendrogram was constructed using MEGA 7 software by the neighbor-joining method [24] with the bootstrap test replicated 1000 times [25].

### 2.2. Plant Growth

The seeds of the Wild-type (WT) tomato (cv. Micro-tom) were used in this work. WT and *SlGRAS10*-RNAi tomato plants were grown in a greenhouse. The plants were grown under the same standard environments as we described previously in our study [23], i.e., 6-h dark (18 °C), 18-h light (25 °C), 60% humidity. Tissues including root, stem, leaves from 4 weeks old WT plants, flowers at anthesis stage, fruits at the immature green, mature green, breaker, breaker plus four days, orange and red stages were collected to perform the gene expression analysis. For each tissue, samples were taken from seven healthy plants. All samples were immediately frozen in liquid nitrogen. Before use, samples were kept at −80 °C temperature.

### 2.3. Vector Construction and Plant Transformation

The 169 bp *SlGRAS10* specific sequences were inserted in the pCAMIBA2301 vector. The pCAMIBA2301 vector was used to construct the *SlGRAS10*-RNAi plasmid. All the primers are shown in Supplementary Table S1. *SlGRAS10*-RNAi plants produced via *Agrobacterium tumefaciens*-mediated transformation. T0 seeds were screened in 1/2 Murashige and Skoog (MS) medium with kanamycin (100 mg L^−1^). To confirm the existence of T-DNA insertions in several lines of *SlGRAS10*-RNAi, genomic PCR was performed. After confirmation, three successful RNAi lines (RNAi-10, RNAi-11, and RNAi-15) in the T2 generation were selected for all analyses. To identify the repression ratio of *SlGRAS10* in RNAi plants, RT-qPCR has been performed.

### 2.4. Measurement of Phenotypic Characterization

To analyze the alterations in plant architecture between WT and *SlGRAS10*-RNAi lines, many parameters including leaf width and length, internode length, and plant height have been measured. Length/width of leaves and internode length were measured from fifteen plants of WT and *SlGRAS10*-RNAi lines. The plant height was calculated 20 days after germination and after that every 7 days. To calculate the flowering time between WT and *SlGRAS10*-RNAi lines, the days to first visible flower and leaf number before the first inflorescence were noted. Fifteen plants from three selected lines of *SlGRAS10*-RNAi and WT were used to measure all the above parameters.

### 2.5. Plant Stress Treatments

For salt stress treatment assay, the roots of the WT tomato seedlings were immersed in 250 mM NaCl solution for 0, 1, 2, 4, 8, 12, 24, and 36 h. The leaves from the stress-treated seedlings were collected. For the dehydration treatment assay, the WT tomato seedlings were softly pulled out from the soil and washed with water, and leaving on dry filter paper at room temperature. The leaves were collected at 0, 1, 2, 4, 8, 12, 24, and 36 h. For the wounding assay, the leaves of WT seedlings were cut with a blade into small pieces and left those leaves on a piece of wetted filter paper in sealed pots for 0, 1, 2, 4, 8, 12, 24, and 36 h at room temperature. For hormonal stress treatment assay, the whole WT tomato seedlings were sprayed, respectively, with water (control), 100 µM abscisic acid (ABA), 50 µM gibberellic acid (GA_3_), or 100 µM 1-aminocyclopropane-1-carboxylic acid (ACC) solution [26], then were sealed off in plastic immediately and left for 0, 1, 2, 4, 8, 12, 24 and 36 h at room temperature. Each sample was frozen in liquid nitrogen immediately and stored at −80 °C until RNA extraction. Each stress experiment was done with three replications.

Homozygous T2 transgenic lines of *SlGRAS10*-RNAi and WT seeds were surface-sterilized and sown on Murashige and Skoog (MS) culture medium. To study the tolerance of seedling development to abiotic stress, transgenic seeds of *SlGRAS10*-RNAi and WT were transmitted to MS medium containing 0 mM (control), 100 mM sodium chloride (NaCl), and 150 mM D-mannitol for 15 days. The seedlings were incubated in a growth chamber under the same conditions described above and measured the root, shoots growth, and seedling weight.

To study the tolerance of seedling development to ABA stress, transgenic seeds of *SlGRAS10*-RNAi and WT were transmitted to MS medium containing 0 mM (control), and 100 mM (ABA) for 15 days. The seedlings were incubated in a growth chamber under the same conditions described above and measured the seed germination rate, root lengths, and shoots lengths of *SlGRAS10*-RNAi and WT.

For the stress experiment, both seedlings of *SlGRAS10*-RNAi and WT were grown in a growth chamber under normal conditions for two weeks. After that seedlings of *SlGRAS10*-RNAi and WT were shifted to a greenhouse for 16 h lights (25 °C) and 8 h darks (18 °C). The one-month-old plants of *SlGRAS10*-RNAi and WT were watered containing 0 (Control), and 200 mM NaCl every 48 h for two weeks to assess the salt tolerance. For drought stress, the one-month-old plants of *SlGRAS10*-RNAi and WT were treated without water for two weeks. For stress-related gene expression analysis, control and stress-treated leaves of *SlGRAS10*-RNAi and WT were harvested. To examine the chlorophyll biosynthesis, gene transcription analysis leaves from one-week-old plants after salt and drought stress experiment were collected. The chlorophyll contents and relative water content (RWC) of *SlGRAS10*-RNAi and WT plants were examined after salt and drought stress treatment. Each stress treatment was completed three times.

### 2.6. Measurement of Chlorophyll and Relative Water Content

To measure the chlorophyll contents, leaf samples were taken from control, salt, and drought stress treated plants and weighed. All leaf samples were randomly chosen from five individual plants. Samples were crushed with liquid nitrogen and extracted with 80% aqueous acetone (10 mL) (*v/v*). With the help of a spectrophotometer, the extract was centrifuged at 4000× *g* for 5 min and the absorbance of the supernatant was noted at 646 and 663 nm. Total Chl (µg mL^−1^) = 20.29A_646_ + 8.02A_663_. According to the method of [27], the measurement of relative water content (RWC) was performed.

### 2.7. Determination of Antioxidant Enzyme Activities, Proline, MDA, and Soluble Sugar Content

Leaves from control, salt, and drought stress treated plants at the same developmental stage were collected to perform the antioxidant enzyme activities, and Proline, Malondialdehyde (MDA), and soluble sugar contents determination. According to the method [28,29], catalase (CAT), peroxidase (POD), and superoxide dismutase (SOD) were determined. According to the manufacturer’s kit protocol (Jiancheng Bioengineering Company Nanjing, China), the superoxide anion radical (O_2_^−^) and H_2_O_2_ content were assayed. Proline contents were determined according to the method [30], total MDA contents were measured according to the method [31], and soluble sugar contents according to the method [32].

### 2.8. Determination of Flavonoid Contents

The flavonoid contents of each sample were determined as described by the Dowd method [33]. Leaves of *SlGRAS10*-RNAi and WT (2 g/sample) were ground in liquid nitrogen and dissolved in 70% ethanol solution and incubated at room temperature for 24 h. 1 mL of extract ethanol solution were mixed with 0.2 mL of 10% (*w*/*v*) AlCl_3_ solution in methanol, 0.2 mL (1 M) potassium acetate and 5.6 mL distilled water. The mixture was incubated for 30 min at room temperature followed by the measurement of absorbance at 415 nm against the blank. The results were expressed as mg/g of quercetin equivalents in milligrams per gram (mg QE/g) of dry extract.

### 2.9. RNA Isolation and Real-Time Quantitative PCR Analysis

Total RNAs were isolated from all collected tissues according to the plant RNA kit (OMEGA BIO-TEK). First-strand cDNA synthesis was determined using a PrimeScript^TM^ RT reagent kit with gDNA Eraser (TAKARA, Kusatsu, Japan). A quantitative Real-Time-PCR (qRT-PCR) was carried out in a Bio-Rad CFX system (Bio-Rad, Hercules, CA, USA) according to the methodology described in [23] using specific primers. Three replicates were used for all samples. *SlUBI* gene was used for normalization. Relative expression levels were calculated based on the 2^−ΔΔCT^ method. All of the qRT-PCR primers are shown in Supplementary Table S1.

### 2.10. Statistical Analysis

Each experiment was conducted with three independent biological replicates. The Student’s *t*-test was used to compare group differences. *p*-values less than 0.05 were considered to be significant.

## 3. Results

### 3.1. SlGRAS10 Is a Member of the PAT1 Subfamily in Tomato

**Figure 1:**
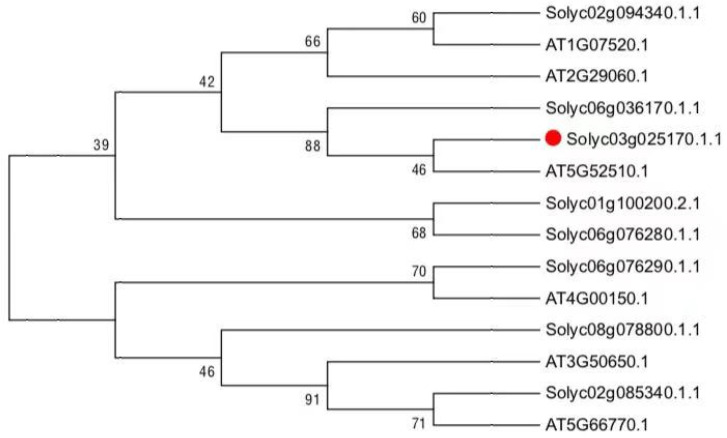
Phylogenetic tree of SlGRAS10 and other known GRAS proteins. A phylogenetic tree of SlGRAS10 and other GRAS proteins was constructed to show the relationship between Arabidopsis and GRAS protein from tomato.

To learn phylogenetic analysis between GRAS family between tomato and Arabidopsis, a phylogenetic tree based on the amino acid sequences was constructed (Figure 1), indicating that the *SlGRAS10* belongs to the PAT1 subfamily and is more closely related to AtSCL8 in *Arabidopsis thaliana* and SlGRAS9 in tomato than to any other proteins among the tree.

### 3.2. Expression Profiles of SlGRAS10 and under Numerous Stress Treatments of Wild-Type Tomato

**Figure 2:**
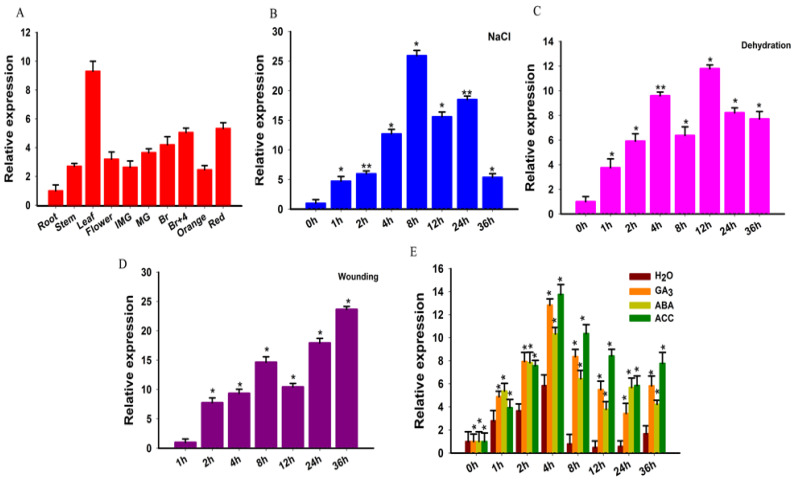
Expression profile analysis of *SlGRAS10* in wild-type tomato plants by quantitative real-time PCR. (**A**) Expression levels of *SlGRAS10* gene in different tissues of wild-type tomato. Root; Stem; Leaf; Flower; IMG, immature green fruit; MG, mature fruit; Br, breaker fruit; Br + 4, 4 days after breaker stage; Orange fruit; Red ripe fruit. (**B**) The expression level of *SlGRAS10* under salt stress. (**C**) The expression level of *SlGRAS10* under dehydration stress. (**D**) The expression level of *SlGRAS10* under wounding stress. (**E**) The response of *SlGRAS10* expression to phytohormones (GA_3_, ABA, and ACC. Asterisks show the significant differences using Student’s *t*-test (* *p* < 0.05, ** *p* < 0.01).

To study the expression profile of *SlGRAS10*, different samples from wild-type tomato plant were collected such as leaves (L), stems (S), roots (R), flowers (F), immature green fruit (IMG), mature green (MG), breaker (Br), breaker + 4 (Br + 4), and red fruit (R). All tissues for expression pattern analysis frozen in liquid nitrogen and kept at −80 °C (Figure 2A). The primers list is given in Supplementary Table S1. The data indicated that the *SlGRAS10* gene expressed in the leaf, breaker + 4, and red fruit, and comparatively lower expression profiles were noticed in other tissues of the tomato plant, indicating its tissue-distinct expression in the tomato plant. The data suggested that *SlGRAS10* may be involved in fruit development. To evaluate the dynamic function of phytohormones in the regulation of various developmental and environmental processes, we studied the expression levels of *SlGRAS10* under different hormonal applications by quantitative PCR.

To study the role of *SlGRAS10* in the stress treatment, the expression profiles of *SlGRAS10* in leaves of tomato seedlings were studied under numerous stress treatments. The *SlGRAS10* showed its higher expression at 8 h and then decreased under NaCl conditions (Figure 2B). The expression of *SlGRAS10* was induced under dehydration treatments and reached a higher value at 12 h (Figure 2C). Under wounding treatments, the expression of *SlGRAS10* showed a higher value at 36 h (Figure 2D). These outcomes propose that *SlGRAS10* might play an important role under abiotic stresses. Assuming that phytohormones are associated with intricate signaling pathways and involved in regulating plant reaction to numerous environmental stresses and developmental processes [34], we further investigated the expression patterns of *SlGRAS10* under several hormonal applications by quantitative RT-PCR. *SlGRAS10* was remarkably induced by ACC application and showed a higher value of 13.7-fold at 4 h, and then decreased but remained at the highest level amongst all hormonal treatments (Figure 2E). The expression of *SlGRAS10* was at a maximum peak at 4 h under the ABA application. For GA_3_, the expression profile of *SlGRAS10* increased distinctly at 4 h and showed a higher value of 12.8- fold. The results of expression levels for *SlGRAS10* showed that the *SlGRAS10* gene may be involved in signal transduction and hormonal stress.

### 3.3. Phenotypic Characterization of SlGRAS10-RNAi Plants

**Figure 3:**
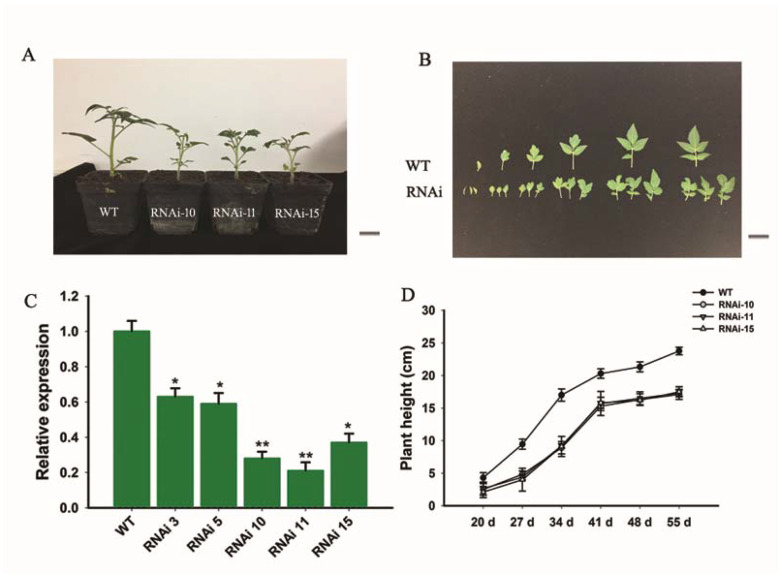
Morphological modifications were displayed by *SlGRAS10* down-regulated transgenic lines. (**A**) The down-regulation of *SlGRAS10* exhibiting decreased plant height. Photos were taken 40 days after sowing. Scale Bar = 1 mm. (**B**) Leaves of the wild-type (upper row) and the *SlGRAS10*-RNAi lines (lower row). Scale Bar = 1 mm. (**C**) The expression levels of *SlGRAS10* in transgenic lines and wild-type. Each value represents the mean ± SE of three independent replicates. Asterisks show the significant differences using Student’s *t*-test (* *p* < 0.05, ** *p* < 0.01). (**D**) The plant height of *SlGRAS10* transgenic three lines and wild-type plants. The plant height was measured every week from 20-day after sowing. Each value represents the mean ± SE of three independent replicates (*n* = 15).

To study the physiological importance of the *SlGRAS10* gene, the transgenic tomato plants were produced by RNAi silencing by transformation with *Agrobacterium tumefaciens*. WT and *SlGRAS10*-RNAi (L10, L11, and L15) plants are shown in Figure 3A. We found five independent transgenic lines, and the expression pattern of *SlGRAS10* was found in all these transgenic lines. The expression patterns of *SlGRAS10* in transgenic lines were reduced as compared to WT (Figure 3C). Three out of five transgenic lines of *SlGRAS10* were selected due to their lowest expression levels and named RNAi10, RNAi11, and RNAi15. These three transgenic lines of *SlGRAS10*-RNAi10, *SlGRAS10*-RNAi11, and *SlGRAS10*-RNAi15 were examined for more phenotypic and molecular investigations.

**Figure 4:**
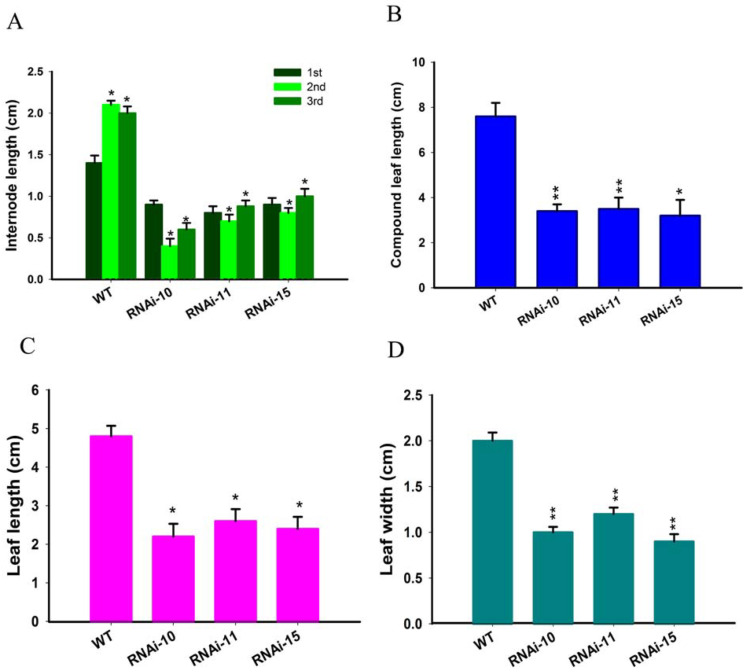
Down-regulation of *SlGRAS10* decreased leaf size and internode length. (**A**) Internode length of the first to third nodes from about 35-days-old *SlGRAS10*-RNAi transgenic and wild-type plants. (**B**) The total mature compound leaf length of *SlGRAS10*-RNAi and wild-type plants. (**C**) The length of the apical leaflet from the mature compound leaves from transgenic and wild-type plants. (**D**) The width of the apical leaflet from the mature compound leaves from transgenic and wild-type plants. The date represents the mean ± SE of three independent replicates (*n* = 15). *, ** indicates a significant difference (*p* < 0.05, *p* < 0.01) between transgenic and wild-type plants.

We noted that selected transgenic lines of *SlGRAS10*-RNAi displayed common phenotypes related to vegetative growth, including short height plants (Figure 3A), and smaller leaves (Figure 3B). We observed and measured the plant height and first three internode lengths of *SlGRAS10*-RNAi (RNAi10, RNAi11, and RNAi15) and WT. The *SlGRAS10*-RNAi plants were remarkably smaller than the height of WT plants (Figure 3D). Similarly, the three internode lengths in three *SlGRAS10*-RNAi lines were smaller than the internode lengths of WT (Figure 4A). The length of compound leaves (Figure 4B), and the length (Figure 4C) and the width (Figure 4D) of mature leaflets were remarkably decreased in three lines of *SlGRAS10*-RNAi compared to WT.

### 3.4. SlGRAS10-RNAi Enhances Tolerance of Seedling Growth to Salt and Drought Stress

**Figure 5:**
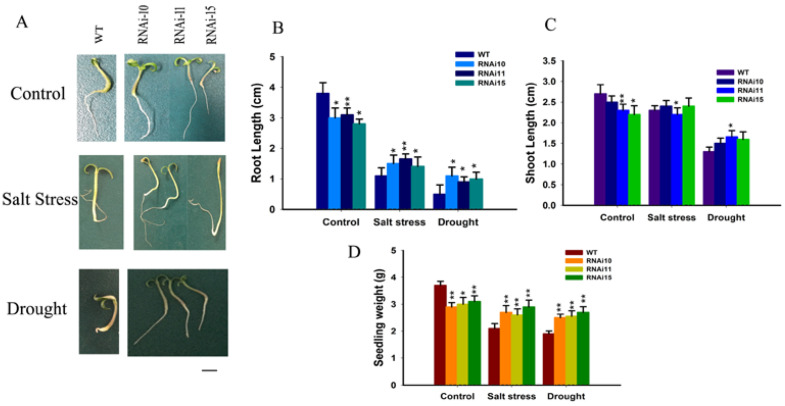
Down-regulation of *SlGRAS10* enhances seedling growth to salt and drought stress. (**A**) The figure shows the growth performance of wild-type and *SlGRAS10*-RNAi-10. RNAi-11 and RNAi-15 seedlings under salt and drought stress after 15 days. Scale bar = 1mm (**B**) Root length of *SlGRAS10*-RNAi three lines and wild-type under control, salt stress, and drought treatments (*n* = 20). (**C**) Shoot lengths of *SlGRAS10*-RNAi and wild-type under control, salt stress, and drought treatments (*n* = 20). (**D**) Seedling weight of *SlGRAS10*-RNAi and after control, salt stress, and drought treatments (*n* = 20). The date represents the mean ± SE of three independent replicates. *, ** indicates a significant difference (*p* < 0.05, *p* < 0.01) between transgenic and wild-type plants.

The substantial stimulation of *SlGRAS10* expression by salt stress and dehydration showed that it might be playing a significant function in salt stress and drought tolerance of tomatoes (Figure 2B,C). At the stage of post-germination, we studied the different influences of salt stress on development between WT and *SlGRAS10*-RNAi tomato seedlings to assess the resistance of WT and *SlGRAS10*-RNAi tomato towards salt stress and drought. Root length was significantly suppressed in WT as well as *SlGRAS10*-RNAi under salt and drought stress, but the root length of *SlGRAS10*-RNAi seedlings was much greater than those of WT (Figure 5B). There is no obvious change in shoot length of WT and *SlGRAS10*-RNAi seedlings under salt stress but the shoot length of WT seedlings was more inhibited under drought stress than those of *SlGRAS10*-RNAi (Figure 5C). The seedling weight of *SlGRAS10*-RNAi was greater than the weight of WT seedlings under salt and drought stresses (Figure 5D). The growth of WT seedlings was more reduced, and the root growth of WT seedlings was significantly inhibited as compared to *SlGRAS10*-RNAi seedlings, showing that silencing of *SlGRAS10* enhances seedling growth more tolerant to salt and drought stress. Furthermore, the seedlings of *SlGRAS10*-RNAi showed more tolerance in fresh weight than WT seedlings when treated with salt and drought stress (Figure 5D).

### 3.5. SlGRAS10-RNAi Enhances Tolerance of Seedling Growth to ABA Stress

**Figure 6:**
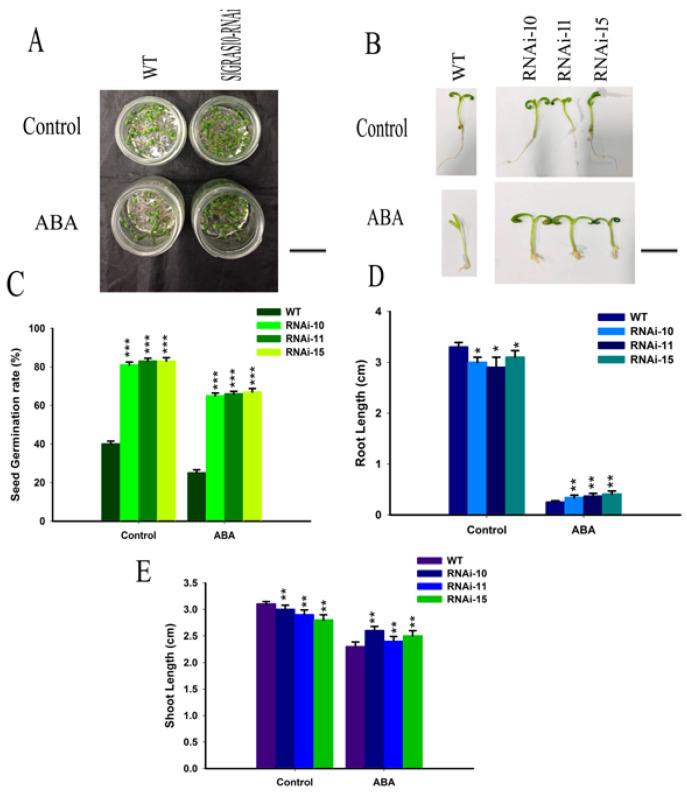
Down-regulation of *SlGRAS10* enhances seedling growth to ABA stress. (**A**) The figure shows the growth performance of wild-type and *SlGRAS10*-RNAi seedlings under control and ABA stress after 15 days. Scale bar = 5 mm (**B**) Photographs of wild-type and *SlGRAS10*-RNAi-10, RNAi-11, and RNAi-15 seedlings under control and salt ABA stress after 15 days. Scale bar = 5 mm. (**C**) The seed Germination rate of *SlGRAS10*-RNAi and after control, and ABA treatments (*n* = 20). (**D**) Root length of *SlGRAS10*-RNAi three lines and wild-type under control, and ABA treatments (*n* = 20). (**E**) Shoot lengths of *SlGRAS10*-RNAi and wild-type under control, and ABA treatments (*n* = 20). The date represents the mean ± SE of three independent replicates. *, **, *** indicates a significant difference (*p* < 0.05, *p* < 0.01, *p* < 0.001) between transgenic and wild-type plants.

The expression levels of *SlGRAS10* are induced by ABA (Figure 2E), it might be playing a significant function in ABA stress. At the stage of post-germination, we studied the ABA stress on the development between WT and *SlGRAS10*-RNAi tomato seedlings to assess the resistance of WT and *SlGRAS10*-RNAi tomato towards ABA stress. The seed germination rate was significantly decreased in WT in response to ABA stress, but the seed germination rate of *SlGRAS10*-RNAi was much greater than that of WT seeds under ABA stress (Figure 6C). Root elongation of both WT and *SlGRAS10*-RNAi was affected in response to ABA stress but the root length of *SlGRAS10*-RNAi was slightly higher than that of the WT under ABA stress (Figure 6D). The shoot lengths of the *SlGRAS10*-RNAi were greater than those of the WT under ABA stress (Figure 6E). Together, these results showed that the seeds and seedlings of *SlGRAS10*-RNAi tolerate ABA stress.

### 3.6. SlGRAS10-RNAi Enhances Tomato Plant Tolerance to Salt and Drought

**Figure 7:**
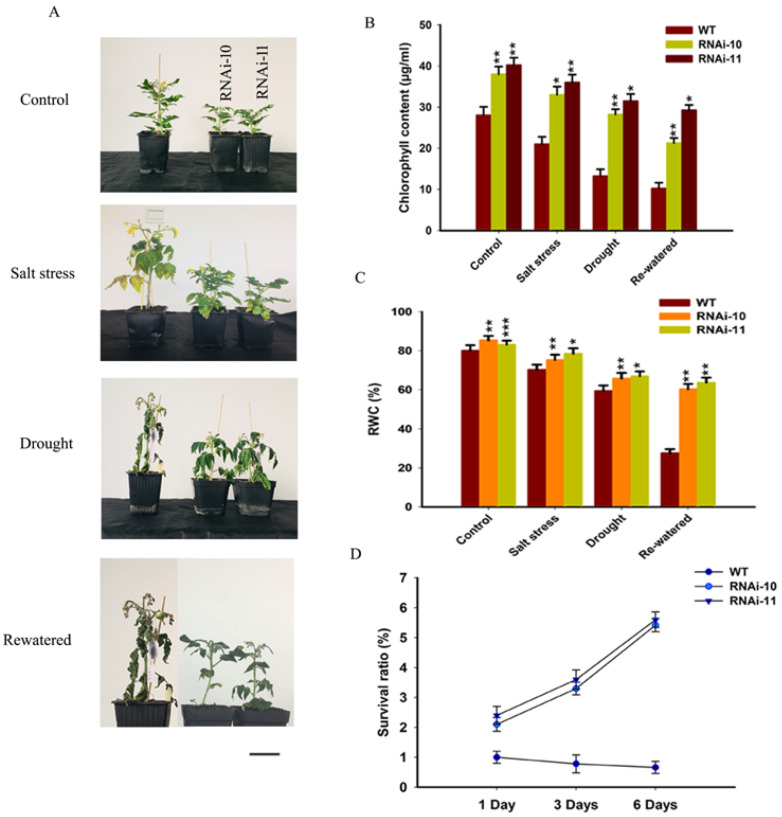
Down-regulation of *SlGRAS10* enhances tolerance to salt and drought stress treatment. (**A**) Photographs of representative plants after one month of control, salt stress, drought stress, and re-watered treatments compared to control plants. Scale bar = 5 mm. (**B**) Total chlorophyll content of *SlGRAS10*-RNAi (L10, L11) and wild-type plants are shown in (**A**) under control, salt, and drought stresses. (**C**) Relative water content (RWC) of *SlGRAS10*-RNAi (L10, L11) and wild-type plants are shown in (**A**) under control, salt, and drought stresses. (**D**) Survival ratio of drought stress plants of WT and *SlGRAS10*-RNAi lines after re-watered at different time points (1 Day re-watered, 3 Days re-watered, and 6 Days re-watered) (*n* = 20). The date represents the mean ± SE of three independent replicates. *, **, *** indicates a significant difference (*p* < 0.05, *p* < 0.01, *p* < 0.001) between transgenic and wild-type plants.

To assess the function of *SlGRAS10*-RNAi lines in response to salt and drought stresses in soil, one-month-old plants of WT and *SlGRAS10*-RNAi (L10, L11) were irrigated with water containing 0 (control), 200 mM NaCl every 48 h for 2 weeks to observe salt stress tolerance. To study drought stress tolerance, one-month-old plants of WT and *SlGRAS10*-RNAi (L10, L11) were deprived of water for up to 2 weeks. The *SlGRAS10*-RNAi (L10, L11) and WT plants showed significant morphological alterations under stress conditions (Figure 7A). All *SlGRAS10*-RNAi plants of L10 and L11 were healthier than WT plants in response to salt and drought stress (Figure 7A). However, *SlGRAS10*-RNAi plants showed slight alterations in their phenotype compared with WT plants under both stresses (Figure 7A). Under salt stress treatment, *SlGRAS10*-RNAi plants showed less necrosis and chlorosis than the WT plants. WT plants showed typical severe desiccation symptoms under drought stress. All leaves of WT plants were wilted, but there are only lower leaves were wilted in *SlGRAS10*-RNAi plants under drought stress (Figure 7A). The results indicated that both stress damage in WT plants was more severe than in *SlGRAS10*-RNAi.

Furthermore, we investigated the total chlorophyll content and relative water content (RWC) in *SlGRAS10*-RNAi and WT plants under control and stresses conditions. The levels of chlorophyll content and relative water content were much higher in *SlGRAS10*-RNAi plants than in WT plants under both stress conditions (Figure 7B,C). Together, these results help to provide a suggestion that the *SlGRAS10*-RNAi confers tolerance under salt and drought stress treatment in tomatoes.

### 3.7. Enhanced Antioxidant Activity in SlGRAS10-RNAi Plants under Stress Conditions

**Figure 8:**
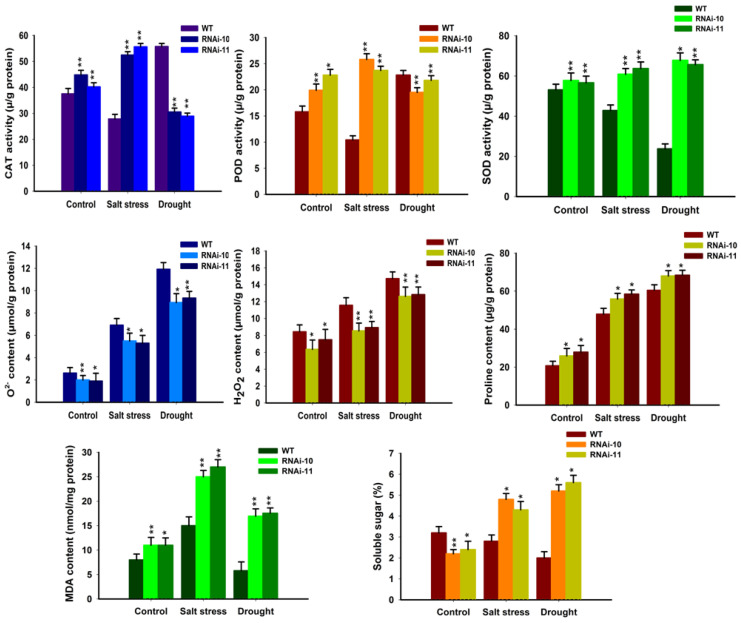
Down-regulation of *SlGRAS10* elevates ROS scavenging ability. CAT, POD, and SOD activities and O^2^^−^, H_2_O_2_, proline, MDA, and soluble sugar contents in the leaves of *SlGRAS10*-RNAi (L10, L11) and WT plants under control and stress conditions. The date represents the mean ± SE of three independent replicates. *, ** indicates a significant difference (*p* < 0.05, *p* < 0.01) between transgenic and wild-type plants.

Salt and drought stresses lead to abundant reactive oxygen species (ROS), which harms membrane structure [35]. It was examined that the alterations in the accretion of O^2−^ and H_2_O_2_ in WT and *SlGRAS10*-RNAi plants under salt and drought stresses. It was observed that less O^2−^ and H_2_O_2_ gathered in *SlGRAS10*-RNAi plants than in WT under control and stress conditions (Figure 8). To study the likely physiological mechanism responsible for the enhanced salt and drought tolerance, we examined the alterations in MDA concentration, soluble sugar contents, and total proline contents from *SlGRAS10*-RNAi and WT plants grown under control and abiotic stress. Total MDA contents were remarkably high in *SlGRAS10*-RNAi plants than WT plants under control and both stress conditions (Figure 8), suggesting that the *SlGRAS10*-RNAi induces potential antioxidative processes preventing significant membrane damage. Under salt and drought stresses, we observed that total soluble sugars were collected more in *SlGRAS10*-RNAi plants than in the WT (Figure 8). Under control and stress conditions, the expression level of total proline contents was higher in *SlGRAS10*-RNAi plants than in WT (Figure 8). We observed that the RNAi plants accrued more proline contents under both stress conditions than control (Figure 8). Moreover, we studied the antioxidants enzyme activities such as SOD, POD, and CAT from *SlGRAS10*-RNAi and WT plants under control, salt, and drought stresses. The expressions of CAT and SOD activities in *SlGRAS10*-RNAi and WT plants were approximately similar under control conditions. For salt stress conditions, the expression pattern of SOD, POD, and CAT were higher in *SlGRAS10*-RNAi than in the WT plants (Figure 8). Under drought stress conditions, the expression pattern of POD and CAT activities in *SlGRAS10*-RNAi plants were down-regulated as compared to WT plants but SOD activity was remarkably higher in *SlGRAS10*-RNAi plants (Figure 8). Overall, these results recommend that *SlGRAS10* may help to enhance the tolerance of transgenic plants to salt and drought stresses.

### 3.8. Up-Regulation of Flavonoid Accumulation in SlGRAS10-RNAi under Stress Conditions

**Figure 9:**
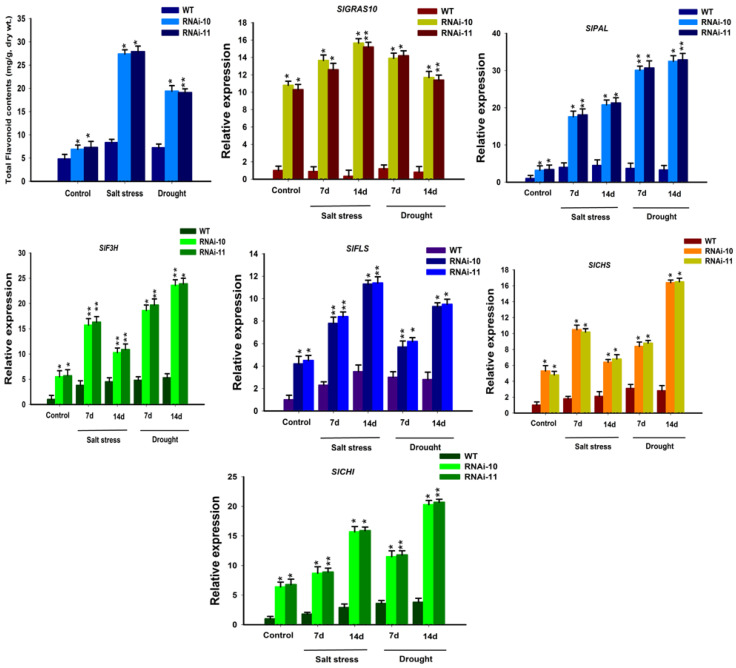
Down-regulation of *SlGRAS10* elevates flavonoid accumulation. Total flavonoid contents and expression profile of flavonoid biosynthesis genes in *SlGRAS10*-RNAi transgenic lines (L10, L11) and WT under control, salt, and drought. The date represents the mean ± SE of three independent replicates. *, ** indicates a significant difference (*p* < 0.05, *p* < 0.01) between transgenic and wild-type plants.

The flavonoid contents were determined in *SlGRAS10*-RNAi and WT plants under control, salt, and drought stress. It was examined that the expression levels of total flavonoid contents in *SlGRAS10*-RNAi plants were greater than the WT plants under control and both stresses (Figure 9). Moreover, the flavonoid biosynthesis pathway genes were examined in the *SlGRAS10*-RNAi and WT plants. The expression patterns of the flavonoid biosynthesis pathway genes (*SlPAL*, *SlF3H*, *SlFLS*, *SlCHS*, and *SlCHI*) were up-regulated in *SlGRAS10*-RNAi compared to WT (Figure 8). The expression of *SlGRAS10*-RNAi plants was significantly up-regulated than the WT plants in response to salt and drought stress (Figure 9). Our data outcomes showed that *SlGRAS10* may induce flavonoid accretion by modulating the flavonoid biosynthesis pathway.

### 3.9. Enhanced Expression of Chlorophyll Biosynthesis-Related Genes in SlGRAS10-RNAi under Stress Conditions

**Figure 10:**
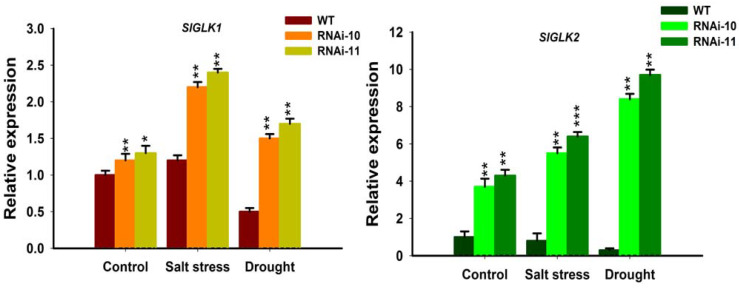
Enhanced expression of chlorophyll biosynthesis-related genes in *SlGRAS10*-RNAi under stress conditions. Expression analysis of *SlGLK1* and *SlGLK2* genes in *SlGRAS10*-RNAi (L10, L11) and WT after control, salt, and drought stress treatments. The date represents the mean ± SE of three independent replicates. *, **, *** indicates a significant difference (*p* < 0.05, *p* < 0.01, *p* < 0.001) between transgenic and wild-type plants.

Alteration of chlorophyll contents in the *SlGRAS10*-RNAi after treatment with salt and drought stress provoked us to examine the influence of the abiotic stresses on chlorophyll biosynthesis at the transcriptional level. Thus, we studied the expressions of both Golden2-like1 (*SlGLK1*) and Golden2-like2 (*SlGLK2*) in leaves of *SlGRAS10*-RNAi and WT plants, suggesting that *SlGLK1* and *SlGLK2* are up regulators of the chlorophyll biosynthesis mechanism. In our results, the relative expressions of *SlGLK1* and *SlGLK2* genes showed higher expressions in *SlGRAS10*-RNAi plants than in the WT under salt and drought stresses (Figure 10). Our results suggest that *SlGRAS10* enhanced the expression of chlorophyll biosynthesis genes by modulating the chlorophyll biosynthesis pathway.

### 3.10. Expression Analysis of Senescence-Associated Genes in SlGRAS10-RNAi under Stress Conditions

**Figure 11:**
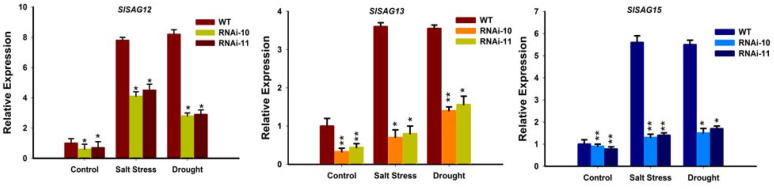
Expression of senescence-associated genes in *SlGRAS10*-RNAi under stress conditions. Expression analysis of *SlSAG12*, *SlSAG13,* and *SlSAG15* genes in *SlGRAS10*-RNAi (L10, L11) and WT after control, salt, and drought stress treatments. The date represents the mean ± SE of three independent replicates. *, ** indicates a significant difference (*p* < 0.05, *p* < 0.01) between transgenic and wild-type plants.

As the Chlorophyll level was altered in *SlGRAS10*-RNAi as compared to WT plants under stress conditions. Thus, we studied the senescence-associated genes *SlSAG12*, *SlSAG13*, and *SlSAG15* in the leaves of *SlGRAS10*-RNAi and WT plants. Leaves from the three-month-old plants of *SlGRAS10*-RNAi lines and WT plants were used for senescence assay. qRT-PCR results showed that the expression levels of the senescence-associated genes in *SlGRAS10*-RNAi lines were down-regulated under control conditions as compared with the WT, indicating that the *SlGRAS10-RNAi* negatively regulates the leaf senescence in tomato (Figure 11). The expression levels of the *SlSAG12*, *SlSAG13*, and *SlSAG15* were slightly higher under salt stress and drought but significantly lower than the levels of the WT (Figure 11). Together, our results suggest a delay in senescence in *SlGRAS10*-RNAi plants.

### 3.11. Expression Analysis of Stress-Related Genes in SlGRAS10-RNAi and WT Plants under Abiotic Stress Conditions

**Figure 12:**
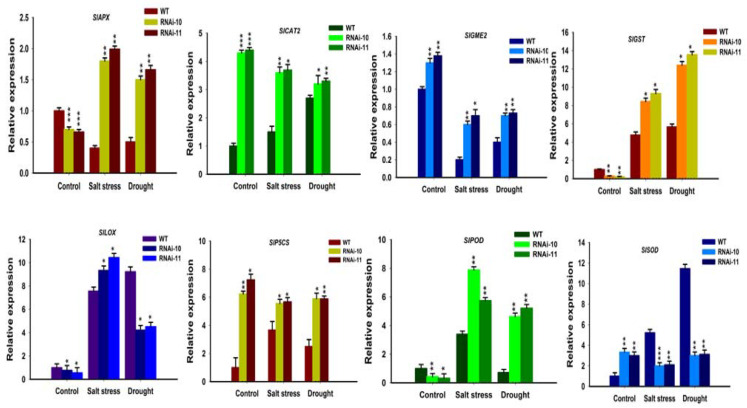
Expression analysis of ROS Scavenging related genes. Expression analysis of stress-related genes (*SlAPX*, *SlCAT2*, *SlGME2*, *SlGST*, *SlLOX*, *SlP5CS*, *SlPOD*, and *SlSOD*) in *SlGRAS10*-RNAi (L10, L11) and WT under control, salt and drought stresses. The date represents the mean ± SE of three independent replicates. *, **, *** indicates a significant difference (*p* < 0.05, *p* < 0.01, *p* < 0.001) between transgenic and wild-type plants.

To study the molecular pathways, the expression levels of the stress-related genes were studied by real-time qPCR in *SlGRAS10*-RNAi lines and WT under salt and drought stress. A key ascorbic acid synthetase (*GME2*), a key proline synthetase (*P5CS*), a catalase (*CAT2*), glutathione S-transferase (*GST*), superoxide dismutase (*SOD*), peroxidase (*POD*), lipoxygenase (*LOX*), and an ascorbate peroxidase (*APX*) were studied for *SlGRAS10* transcription factor. The relative expressions of *SlAPX* and *SlGST* genes showed higher expressions in the *SlGRAS10*-RNAi plants than the WT under salt and drought stress (Figure 12). The *SlPOD* gene indicated higher expression levels in *SlGRAS10*-RNAi plants than in WT after both stress treatments (Figure 12). *SlCAT2* and *SlP5CS* were up-regulated after control conditions in *SlGRAS10*-RNAi plants, but after salt and drought stress treatment, the expression levels of *SlCAT2* and *SlP5CS* were significantly reduced in WT than *SlGRAS10*-RNAi plants (Figure 12). The *SlLOX* gene indicated higher expressions under salt stress conditions in *SlGRAS10*-RNAi plants compared to WT (Figure 12). Together, our results showed that *SlGRAS10* may play role in inducing the gene expression which is involved in stress signaling pathways in tomato.

## 4. Discussion

Environmental stresses such as salt and drought are primary issues that considerably decrease agricultural yield. The progress in stress tolerates crops will be significantly helpful for current agrarian zones which are likely to abiotic stresses. Plants come across a series of stresses, including drought, salinity, cold, heat, and light so on. Such kinds of environmental stresses badly affect the growth, productivity, and survival ratio of plants [36]. Plants improve their numerous mechanisms and pathways to oppose these stress conditions, which allow them to take on under such environments [37]. Molecular plant biology techniques allow us to determine numerous genes, such as transcription factors which play dynamic functions in temporal and spatial genes expression under stress tolerance which might be caused by one or more biotic and abiotic stresses [38]. From the GRAS protein family in tomato, eleven proteins belong to the PAT1 branch. In this study, the *SlGRAS10* gene from the PAT1 branch is functionally described. Our study elucidated the function of a *SlGRAS10* in tomato confers abiotic stress tolerance. The tissue expression pattern showed that *SlGRAS10* was mainly found in leaves. *SlGRAS10* was responsive to salt stress (NaCl), dehydration, wounding, and hormonal treatment (GA_3_, ABA, and ACC) (Figure 2). Our results suggested that *SlGRAS10* may be intricate in leaf growth, abiotic stress-responsive, and mediate hormone signaling.

Down-regulation of *SlGRAS10* significantly reduced the plant height (Figure 3A), leaf size as well as internode lengths of tomato (Figure 4A,C,D). In our previous study, overexpressing plants of *SlGRAS7* enhanced tolerance to abiotic stress [23]. In the present work, physiological, morphological, and molecular data confirmed that the *SlGRAS10* gene confers salt and drought stresses. We produced tomato trans-genetic plants with down-regulation of *SlGRAS10* by the RNAi method and showed comprehensive physiological studies. First, we examined the seedling growth of *SlGRAS10*-RNAi and WT under salt and drought stress and found that *SlGRAS10*-RNAi enhances seedling growth more tolerate under salt and drought stress than WT (Figure 5A). Our results demonstrated the down-regulation of *SlGRAS10* plants more tolerant under salt and drought stress. The immersion of *SlGRAS10* tomato plants in response to abiotic stress proved that down-regulation of *SlGRAS10* conferred the tomato plants with greater tolerance and responsiveness under salt and drought stress than WT. Therefore, these results appeared to be a positive relationship between the *SlGRAS10* and abiotic stress in RNAi lines.

ABA plays a vital role in response to abiotic stress, especially in water-deficient environments, where plant species elevate the cellular level of ABA to combat drought stress. Under drought stress, *NCED3* is a dominant contributor to ABA production in *Arabidopsis*. ABA regulates the dormancy and germination of the seed, inhibiting root growth in response to salinity and drought stresses [39,40]. In our study, we examined the biological parameters in *SlGRAS10*-RNAi and WT seedlings under control and ABA stress. The *SlGRAS10*-RNAi seedlings showed a higher germination rate as compared to WT in response to ABA stress (Figure 6). We observed that the root and shoot lengths of WT seedlings are much lower than those of the *SlGRAS10*-RNAi seedlings. Thus, these results lead to an indication of a positive relationship between the *SlGRAS10* and ABA stress in RNAi-lines.

Environmental stresses generally cause several biochemical and physiological modifications in plants. Therefore, we more examined the physiological base responsible for the modifications in tolerance of *SlGRAS10*-RNAi and WT plants to salt and drought stress. For example, chlorophyll, MDA, and RWC contents are common parameters for assessing abiotic stress resistance and tolerance in plants [41,42,43,44]. In this work, we examined the direct relationship between modifications in *SlGRAS10* expression and the biological parameters related to stress tolerance. The *SlGRAS10*-RNAi plants showed less chlorosis and necrosis than in WT plants after treated with salt, drought stress and after re-watered drought plants (Figure 7A). We examined that the *SlGRAS10*-RNAi plants collected more chlorophyll contents than WT plants under abiotic stress and re-watered drought plants (Figure 7B). Moreover, water contents in *SlGRAS10*-RNAi plants were higher than WT plants under salt, drought stresses, and re-watered drought plants (Figure 7C). Moreover, *SlGLK1* and *SlGLK2* (chlorophyll biosynthetic genes) showed higher expression in *SlGRAS10*-RNAi lines under control, and both abiotic stresses (Figure 10). Together, these outcomes help to support the argument that the increased tolerance of *SlGRAS10* down-regulated plants is associated with more accretion of chlorophyll and RWC contents.

Abiotic stresses generally induce accretion of ROS and lipid peroxidation which lead to oxidative stress [45]. MDA is a decomposition product of polyunsaturated fatty acids and has been commonly used as a parameter for lipid peroxidation [46]. In this study, we examined that *SlGRAS10*-RNAi lines showed more accretion of MDA contents than WT plants when treated to salt and drought stress (Figure 8). This might be because of the protective function of antioxidant enzymes in *SlGRAS10*-RNAi lines, suggesting that the synergistic effect of various ROS-scavenging enzymes can effectively improve the antioxidant capacity of plants, thereby reducing the degree of oxidative stress in *SlGRAS10*-RNAi lines. Thus, these results suggest that *SlGRAS10* may confer more tolerate towards oxidative stress in plants. We believed that the down-regulation of *SlGRAS10* improves the ability of transgenic plants to scavenge reactive oxygen species. Antioxidant enzymes such as CAT, POD, and SOD reduce oxidative and osmotic deterioration to cellular enzymes, plasma membrane proteins, and integrity in plants [35,47]. The down-regulation of *SlGRAS10* plant lines showed more accumulation of proline and total soluble sugar contents than WT towards salinity and drought stress (Figure 6). *SlGRAS10*-RNAi lines showed a higher accumulation of H_2_O_2_ and O^2−^ contents than the WT plants after treated to both stresses (Figure 8). Probably, soluble sugar and proline contents secure *SlGRAS10*-RNAi against ROS, reducing oxidative loss. ROS scavenging enzymes, for example, POD, SOD, CAT, GST, and APX reduce oxidative loss and increase abiotic stress tolerance in plants [48]. Here, the expression levels of CAT and POD were higher in *SlGRAS10*-RNAi plants than in WT under control and salt stress treatments (Figure 8), and the SOD activity in *SlGRAS10*-RNAi plants was up-regulated under control and both stresses (Figure 8). These results showed that the ROS scavenging ability in *SlGRAS10*-RNAi plants was greatly improved and the down-regulation of *SlGRAS10* in tomato plants confers abiotic stress tolerance. Plants are often affected by biotic and abiotic environments, and therefore they have developed distinguished physiological and developmental pathways by gene expression regulation under biotic and abiotic stresses [49]. These results showed that the expression of stress-related genes such as *SlAPX*, *SlGST SlPOD*, *SlCAT2*, *SlGST SlP5CS,* and *SlLOX* genes in *SlGRAS10*-RNAi plants was much higher under both stress conditions than in WT (Figure 12). Thus, we examined that the increased tolerance to abiotic stress is primarily attributable to considerably higher expression levels of stress-responsive genes. Together, the expression level alterations of stress-related genes showed a dynamic regulatory function of the *SlGRAS10* under abiotic stress environments, and certainly influence salt stress tolerance.

Flavonoids belong to a group of secondary metabolites that have the distinct antioxidant capability in plants [50]. These secondary metabolites can improve plant tolerance under abiotic stresses because of their ability to reduce free radicals, peroxides, superoxide which were accumulated during stress conditions [51,52]. Therefore, we supposed that the accretion of secondary metabolites (flavonoids) can increase tolerance to oxidative and osmotic stress. It was determined that down-regulation of *SlGRAS10* plants accumulates more flavonoids, as well as the flavonoid biosynthesis pathway genes, showed higher expressions in *SlGRAS10*-RNAi lines in response to salt and drought stress conditions than in WT (Figure 9). These findings suggest that up-regulation of flavonoid accumulation leads to enhanced tolerance to oxidative stresses.

Therefore, together our study provides evidence that the down-regulation of *SlGRAS10* confers abiotic stress tolerance in tomatoes. The higher expression levels of flavonoid biosynthesis genes, chlorophyll biosynthesis genes, and ROS scavenging genes may cause an increase in the capability of *SlGRAS10*-RNAi tomato plants to deal with related stresses.

## 5. Conclusions

In summary, our present work concerned the physiological and morphological aspects of *SlGRAS10*-RNAi transgenic tomato under abiotic stress environments, including molecular mechanisms for expression levels of stress-related, chlorophyll, senescence-associated, and flavonoid biosynthesis genes. We concluded that the down-regulation of the *SlGRAS10* gene improves tolerance to abiotic stresses by increased chlorophyll contents, flavonoid accumulation, and ROS scavenging enzymes. Therefore, these physiological alterations improve and enhance the tomato plant’s ability to sustain under abiotic stress environments. Our work provides evidence of GRAS functions against abiotic stresses as and helps to enhance our knowledge about their functions under abiotic stresses.

## Data Availability

No datasets were generated and analyzed for this study.

## Supplementary Materials

The following are available online at https://www.mdpi.com/article/10.3390/genes12050623/s1, Table S1: List of primers used in cloning and qRT-PCR.
